# The not so hidden impact of interictal burden in migraine: A narrative review

**DOI:** 10.3389/fneur.2022.1032103

**Published:** 2022-11-03

**Authors:** Maurice Vincent, Lars Viktrup, Robert A. Nicholson, Michael H. Ossipov, Bert B. Vargas

**Affiliations:** ^1^Eli Lilly and Company, Indianapolis, IN, United States; ^2^Evidera, Bethesda, MD, United States

**Keywords:** interictal burden, MIBS-4, migraine, quality of life, disability, co-morbidities, headache, pain

## Abstract

Migraine is a highly prevalent neurological disease of varying attack frequency. Headache attacks that are accompanied by a combination of impact on daily activities, photophobia and/or nausea are most commonly migraine. The headache phase of a migraine attack has attracted more research, assessment tools and treatment goals than any other feature, characteristic, or phase of migraine. However, the migraine attack may encompass up to 4 phases: the prodrome, aura, headache phase and postdrome. There is growing recognition that the burden of migraine, including symptoms associated with the headache phase of the attack, may persist between migraine attacks, sometimes referred to as the “interictal phase.” These include allodynia, hypersensitivity, photophobia, phonophobia, osmophobia, visual/vestibular disturbances and motion sickness. Subtle interictal clinical manifestations and a patient's trepidation to make plans or commitments due to the unpredictability of migraine attacks may contribute to poorer quality of life. However, there are only a few tools available to assess the interictal burden. Herein, we examine the recent advances in the recognition, description, and assessment of the interictal burden of migraine. We also highlight the value in patients feeling comfortable discussing the symptoms and overall burden of migraine when discussing migraine treatment needs with their provider.

## Introduction

Migraine is an especially common disorder, with a prevalence exceeding that of diabetes, epilepsy and asthma combined, affecting as much as 15% of the population of the United States (US) (1, 2). Migraine is the second leading cause of years lived with disability and the leading cause among adult women less than age 50 (3) and can place significant burden on an individual's ability to function at their best at work, home, and social activities. In spite of its prevalence, recognition of migraine as an important disabling public health concern has been slow in coming, and it was not included in the Global Burden of Diseases, Injuries, and Risk Factors (GBD) studies prior to 2000 (4).

Although migraine has been traditionally regarded as a paroxysmal disorder characterized by headache attacks separated by normal intervals, patients are often affected during headache-free phases (5). Data on hypersensitivity to external stimuli outside attacks and the migraine interictal impact on quality of life became available around 3–4 decades ago (6–9). Guidelines for clinical trials evaluating the benefit of migraine preventive treatments make either change in migraine days, moderate-severe headache days, or responder rate, the recommended primary endpoint (10, 11). Although a focus on the ictal symptoms and frequency is helpful for diagnosing migraine and evaluating the benefit of a treatment and evaluating a standard outcome, migraine involves much more than the headache attack. Guidelines for evaluating migraine preventive treatments also recommend including a measure of health-related quality of life (QoL) and/or disability as a secondary outcome and yet a recent evaluation of clinical trials for migraine and other headache found that only 40.3% included a patient reported outcome measure of disability/impact/HRQoL (12). Thus, it appears that many clinical interactions for migraine focus on symptoms and/or counting monthly migraine days and may be missing a substantial part of the picture by overlooking the impact of migraine on QoL during and also in between the pain phase of migraine attacks.

**Figure 1:**
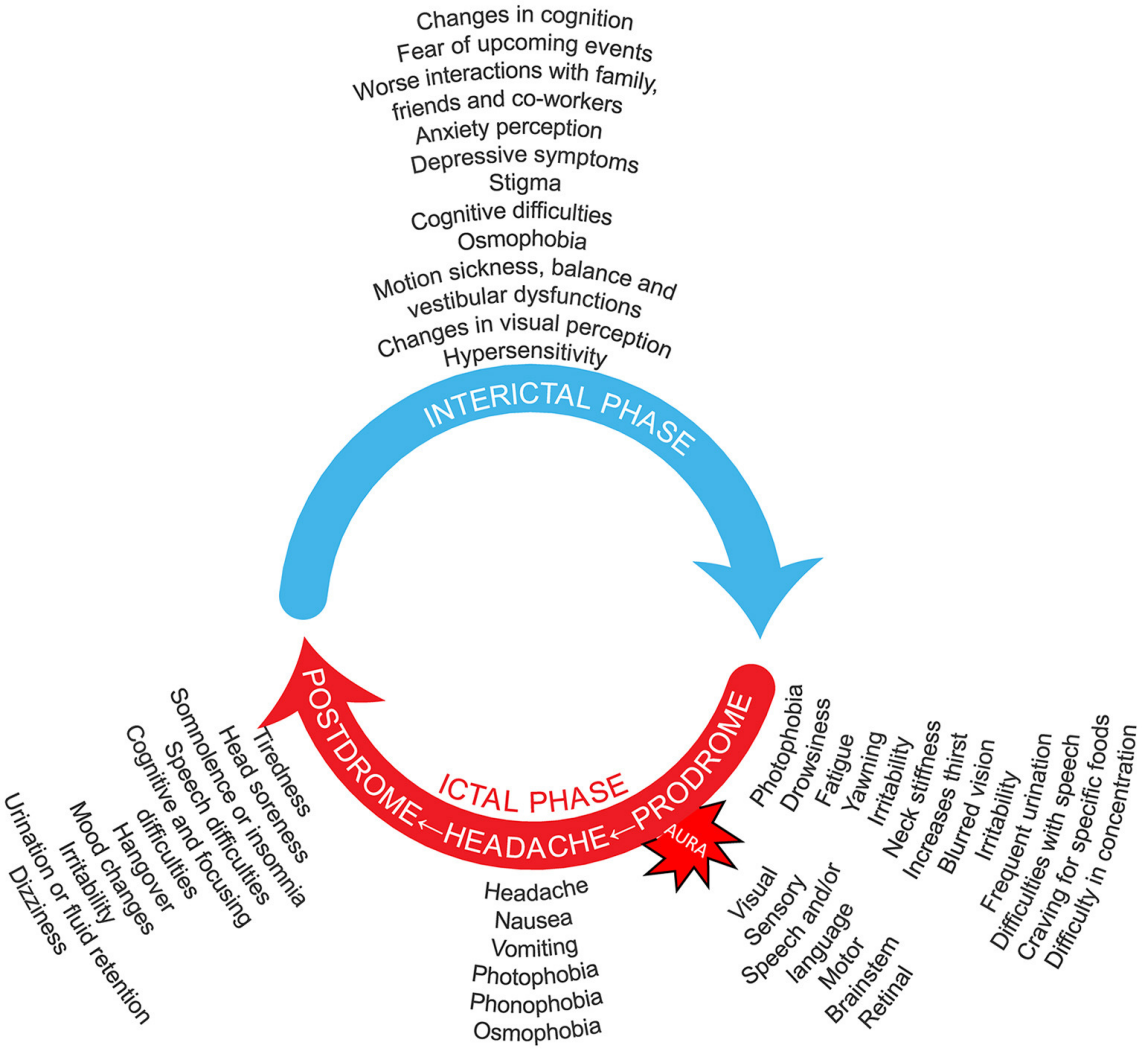
Migraine is an episodic, cycling neurologic condition, where the patient with migraine goes through cycles of relative quiescence (interictal period) that are punctuated by occasional attacks that include the migraine headache. Thus, patients with migraine cycle through an interictal period, that leads to the premonitory phase, then the full-blown migraine attack that includes the severe migraine headache, that is in turn followed by a postdrome phase of waning symptoms and fatigue. Symptoms listed for the interictal (5, 13–26), prodromal (27–32), headache (27, 29, 30, 32–35), and postdromal (27, 29, 32–38) phases, as well as the migraine aura symptoms (27), are suggestions not based on frequency and do not include all possibilities. It should be noted that no phase is obligatory in the migraine wheel; and not all possible symptomatology is depicted. Many symptoms may be present around the entire cycle. One may imagine that the speed with which the migraine wheel rotates distinguishes episodic from chronic migraine. Preventive treatment is a break that reduces this rotation speed. Acute treatment does not reduce that speed but hides one of its components.

In addition to the headache phase, migraine can be accompanied by a constellation of other manifestations in varying combinations apart from the symptomatology listed in the diagnostic criteria, including visual disturbances, osmophobia, allodynia (i.e., as the normally non-noxious stimulus from light touch or brushing of the skin causing pain or discomfort), pain on movement, motion sickness, vestibular dysfunctions, cognitive symptoms, and cranial autonomic symptoms (Figure 1). Patients with migraine may experience many of these symptoms even in the interictal phase, although generally at a reduced frequency and/or intensity (13–20).

Often, some of these symptoms can impact quality of life (QoL) during and in between attacks. Between attacks patients may be fearful, anxious, or worried about when their next one might occur or reflect a patient's concerns about how a future attack can affect plans or activities (21, 39, 40). The impact produced by these phenomena has been described as Interictal Burden (IIB) (5). Moreover, patients with migraine may be subjected to social stigma, which inhibits their seeking treatment and adds to the emotional burden of the disease (41). These non-headache aspects of migraine can be disabling in their own right. We suggest that simply measuring a change in the number of days with a headache may be an inadequate measure to gauge the true impact of migraine, as well as the success of a novel treatment, and that a more holistic approach should be added to future investigations to fully capture the potential benefit to a patient's overall wellbeing.

The need for objective data, such as monthly headache days, by third-party payers in order to approve access to certain therapies, in addition to hesitancies in patient-provider communication or dialogue may be partially responsible for QoL not receiving the attention that it may warrant. It is possible that closed-ended questions constrain discussion of how or why a patient is presenting and what they are currently doing and/or taking that is not working due to the impact it has on their life. If IIB is discussed or brought up by the patient, then that would help identify a need for initiating or modifying treatment. For instance, in a study of doctor-patient interactions, the investigators found that “characteristically, after a brief period of time (mean, 18 s), and most often after the expression of a single stated concern, the physicians in our study took control of the visit by asking increasingly specific, closed-ended questions that effectively halted the spontaneous flow of information from the patient” (42). This is unfortunate because migraine is a condition in which provider-patient communication is paramount especially since the clinical and/or neurological examinations of patients with migraine are typically normal and providers are unable to rely upon biomarkers to aid in diagnosis or tracking of disease progression. This underscores the importance of the patient's narrative which should be explored along with their ideas, feelings, and expectations, which would provide new insights into the illness as the patient is experiencing it (43). Exploring interictal burden necessitates encouraging patients to talk about the entire migraine experience. The use of open-ended questions and an “ask-tell-ask” strategy can yield important information about IIB and QoL and may also leads to shorter office visits, more frequent discussions of preventive therapy, and higher levels of satisfaction for both patient and health-care provider (39).

## Migraine frequency

Migraine can be classified based on headache frequency. Chronic migraine (CM) is defined by a patient having ≥15 headache days/month for >3 months with at least 8 of which fulfill diagnostic criteria for migraine (27). There is a proposed definition of episodic migraine (EM) as being “Headache occurring on <15 days a month over the last 3 months, which on some days is migraine” (44). Attempts are being made to identify high-frequency episodic migraine (HFEM) and low-frequency episodic migraine (LFEM), in order to understand how migraine frequency may affect potential responses to medications, and to help in the development of treatment guidelines, as well as to better understand the influence of frequency on QoL (45–50). Although QoL is underutilized as an indicator of disease severity and response to treatment, we are certainly not the first to underscore its importance in making treatment decisions. The American Migraine Prevalence and Prevention (AMPP) Advisory Group proposed that preventive treatment be offered to patients with ≥6 monthly headache days regardless of impairment, those with ≥4 monthly headache days with “some impairment,” or ≥3 monthly headache days with severe impairment or bed rest (51). It was also proposed that preventive treatment be considered for patients with 4 or 5 monthly headache days and no impairment, 3 monthly headache days and some impairment, or 2 monthly headache days and severe impairment, whereas it is not indicated for patients with <4 monthly headache days and no impairment, or ≤1 monthly headache days regardless of impairment (51). These recommendations were also made within the most recent version of the American Headache Society's consensus statement (52). Data suggests that IIB is an important metric for organizations to add to future guidelines, consensus statements, and recommendations for initiating treatment and evaluating response to treatment. Using MHDs and IIB, as part of overall QoL considerations, to guide preventive treatment decisions, might not only improve the patient's overall QoL, but may help reduce the risk of a patient progressing from EM to CM (33, 53, 54).

## Burden of migraine–Migraine attacks

The burden of migraine extends beyond disability-adjusted life years (DALYs) and years lost to disability (YLDs), as defined by the Global Burden of Disease (55). The debilitating symptoms can affect daily functioning not just during a migraine attack, but extend from the prodrome phase through the postdrome phase (56). In a recent cross-sectional, multi-country, online survey of participants who self-reported a migraine diagnosis, 54% reported severe disability related to migraine, and 30% reported that they restricted their activity for 1–2 days during an attack (56).

The headache phase of a migraine attack is but a part of the overall migraine experience, as illustrated in Figure 1. The headache phase can be preceded by up to 72 h by a prodrome phase, also known as premonitory phase, that may consist of some combination of fatigue, difficulty in concentrating, neck stiffness, sensitivity to light and/or sound, nausea, blurred vision, yawning, and pallor, among other phenomena (1). The early onset of these premonitory symptoms before a migraine headache phase suggests that changes in CNS activity precede the onset of the migraine headache phase (57). A substantial proportion of patients can predict a migraine headache from the presence of prodromes with a reasonable degree of accuracy, even hours before the headache phase onset (58). Most of the premonitory symptoms continue, and may intensify, in the headache phase, suggesting that the premonitory phase symptoms may signal an increase in the neurophysiologic changes preceding the pain phase of an attack (58).

The headache phase (4–72 h) may be followed by a postdrome (i.e., postictal) phase that can last up to 24 h (59). This phase has not been well-studied (27, 36). The most commonly cited symptoms of the postdrome phase are sleepiness/weariness or feeling tired, stiff neck, difficulty concentrating, and mild residual head discomfort (27, 36, 60). The postdrome phase is not trivial, as many patients report that they are “somewhat limited,” and a majority (63%) of patients with chronic migraine are “very/extremely limited,” in completing daily activities (36, 56). The interictal phase constitutes the time between the attacks. Allodynia, is an important symptom associated with migraine, yet not mentioned in the International Headache Society International Classification of Headache Disorders, 3^rd^ edition (IHS ICHD-3) classification of migraine (27). This symptom can manifest as discomfort when combing or shaving, or when wearing glasses, contact lenses, earrings, a hat, or even tight clothing, and thus can impact activities of daily living (34). Many studies have addressed allodynia in migraine with respect to the ictal phase (33, 61–64). Whereas, interictal allodynia and hypersensitivity have been documented (16, 17), a concerted effort is needed to better understand the extent to which this is happening and whether it contributes to the burden between attacks. Studies bringing this issue to a better level of evidence are lacking. Here is exactly where the importance of narrative medicine becomes apparent.

## Burden of migraine–Interictal period

Patients with migraine have a burden of disease that likely extends into the interictal phases, impacting quality of life even between migraine attacks (65). Moreover, many symptoms that are associated with the ictal phase of migraine can still be detected interictally, although, in general, less frequently and with less intensity than during an active migraine attack. However, this phase of migraine has received scant attention until recently. The IIB in people with migraine impacts overall activity, with lower levels of mobility, as well as a greater level of sleepiness and reduced vigor when compared to those who do not have migraine (65). A cross-sectional study of patients who had migraine without aura found that there was an association between executive disturbances and the duration and intensity of migraine headache as well as evidence of mild executive dysfunction during the interictal phase (40). Emerging literature suggests interictal symptoms may involve both emotional and non-emotional (“neurological”) symptoms (13, 14, 39, 64, 66).

## Functional impact

A prospective, longitudinal, Web-based survey of 13,064 respondents with migraine [Chronic Migraine Epidemiology and Outcomes (CaMEO) Study] found that migraine had a significant impact on many important aspects of life that reaches beyond the individual attacks such as marital, parenting, romantic and family relationships, career/financial achievement and stability, and overall health. The reported burden was consistently greater among patients with chronic migraine compared to those with episodic migraine, and there were few sex differences (66). The ObserVational survey of the Epidemiology, tReatment and Care of MigrainE (OVERCOME) study found, in both the US and Japan, headache frequency was associated with increased disability and/or absenteeism, and that regardless of frequency, patients with migraine experienced substantial impacts on productivity and QoL (67).

## MIBS-4

Lipton, Buse and colleagues developed the Migraine Interictal Burden Scale (MIBS)-4 to quantify the interictal burden over the past 4 weeks on days without a headache. This self-administered questionnaire consists of 4 items measuring impairment in work or school, impairment in family and social life, difficulty making plans or commitments, and emotional/affective and cognitive distress (39, 68). Each question was scored by the patient to give a total MIBS-4 score (score range 0–12; 0 = none and ≥5 = severe). Moderate correlation validity was observed between MIBS-4 and health-related QoL, lost productivity and psychological disorders, but also ictal disability (39). Though, further development of interictal scales may be desirable, the MIBS-4 has shown usefulness in both real-world evidence (RWE) studies and as an additional tool in pharmaceutical migraine studies. When MIBS-4 was applied in a cross-sectional, observational, population-based web survey (OVERCOME-Japan) of Japanese people with migraine, 41.5% of respondents experienced moderate-to-severe interictal burden that worsened with increasing frequency (67). A cross-sectional survey of 10 European Union countries (Eurolite survey) revealed that patients with migraine suffered from interictal anxiety that increased with headache frequency and intensity, as well as interictal avoidance behavior (69). Patients reported that they felt that they had done less well in their education, careers, or earnings because of migraine. About 10% worried about their next headache and felt that family and friends did not understand their burden. A recent pharmaceutical study reported that treatment with the calcitonin gene-related peptide (CGRP) monoclonal antibody galcanezumab significantly reduced the interictal burden of migraine as measured by MIBS-4 (70, 71). Interestingly, among its sample of more than 60,000 individuals with migraine, OVERCOME (US) found that, in a study that utilized machine learning to determine what factors, among more than 50 sociodemographic, clinical, and migraine-related factors were most associated with seeking care for migraine, that higher IIB was the factor most associated with differentiating those who did/did not seek care for migraine (72). According to the Clinicaltrials.gov website 4 clinical trials for migraine are either ongoing (1) or recently ended (3) that included a consideration of IIB. Thus, the MIBS-4 is a useful scale for the assessment of the interictal burden in migraine that should be more widely used. Moreover, it is the authors' hope that additional, and better, instruments be developed for assessment of the interictal burden in migraine, both in practice and as a research tool.

## Non-emotional (“neurological”) symptoms: Physiologic and neuroimaging changes

It has been well-appreciated that patients with migraine can experience hypersensitivity to light, sound, and odors during the interictal phase. In a study that examined discomfort threshold levels to auditory (13, 14) or visual (14) stimuli, patients with migraine were found to have significantly greater sensitivity to light and sound during the interictal phase when compared to healthy control subjects. Studies assessing auditory and visual evoked potentials indicated enhanced interictal activation of the brainstem and visual cortex in patients with migraine (73, 74). Patients with migraine who show interictal photosensitivity were found to have thicker cortical regions (i.e., right lingual, isthmus cingulate and pericalcarine regions, and the left precentral, postcentral and supramarginal regions) (75). In addition to photophobia, patients with migraine may have persistent, continuous visual disturbances, such as “visual snow” (20). A study was conducted where patients with migraine were presented with visual stimuli and subjected to functional magnetic resonance imaging (fMRI) (76). This study found that patients with migraine had enhanced cortical responsiveness to visual cues during the interictal period. Other studies found that patients with migraine may have interictal osmophobia, and that higher olfactory sensitization may be associated with a higher burden of disease (18, 19). Patients with episodic or chronic migraine may also have enhanced levels of cortical excitability during the interictal phase compared to normal control subjects that contributes to sensory hypersensitivities (77, 78), as well as interictal autonomic abnormalities (22).

Patients with migraine also show interictal vestibular symptoms of dizziness and vertigo (23). Participants with migraine underwent fMRI while watching customized forward self-motion roller coaster videos on a screen, and rating their perceptions of dizziness and motion sickness during the interictal phase (23). Changes in activity of brain regions (inferior and superior occipital gyrus, middle frontal gyrus, pontine nuclei, and cerebellar lobules V, VI, and VIIb) correlated with motion sickness and disability scores, suggesting an increased susceptibility to dizziness and motion sickness (23). It has been suggested that there are common mechanisms and neurologic pathways that contribute to symptoms of motion sickness and of migraine (24).

The concept of interictal allodynia has been proposed and examples of interictal allodynia do exist (16), as do reports of enhanced sensitivity to pain (17, 64, 79). However, a high-level evidence is not yet available, and this has not been rigorously studied. If interictal allodynia were to be found to indeed exist, though, it would be a significant contributor to IIB much as ictal allodynia is a contributor to the burden of the migraine attack. This symptom calls for additional attention.

The concept that patients with migraine have a hyperexcitable, or sensitized, cortex brings to mind some features of migraine which are similar to those of epilepsy. Both conditions are episodic, disorders where a susceptible brain is hyperexcitable and may be associated with abnormal neuronal activity (80). It is an incorrect presumption that patients with epilepsy are only affected during seizures and “normal” in between; rather, many individuals with epilepsy are not truly “normal” interictally, even if seizures are controlled. Likewise, what differentiates migraine patients from individuals without migraine is the permanent susceptibility to an attack, independently from the presence of triggers. Thus, like patients with epilepsy, those with migraine are likewise impacted during the interictal phase.

Some studies have shown that patients with episodic migraine (81, 82) or chronic migraine (83, 84) have elevated blood or saliva levels of CGRP interictally. In one study, the elevation in CGRP levels in patients with chronic migraine was not significantly different from levels in control patients, and the elevations in CGRP levels of patients with CM were significantly greater than those of patients with EM (83). Moreover, in one study, the interictal levels of CGRP of patients with chronic migraine are significantly elevated relative to those of patients with episodic migraine, whereas those levels, though elevated relative to control individuals, were not significantly so (83). Moreover, patients with chronic migraine who were responsive to treatment with onabotulinumtoxin A had reduced interictal CGRP blood levels relative to those who were not responsive to the treatment (85, 86).

## “Objective” interictal findings

Few studies have assessed potential changes in the neurophysiology during the interictal phase. One study using fMRI in 32 patients with migraine during the interictal phase found altered global sensory processing in the pain-free state, providing a neurophysiological basis for a potentially altered auditory, gustatory, motor and somatosensory processing (87). A proton magnetic resonance spectroscopy (1H-MRS) and fMRI daily in one patient for 21 days identified interictal abnormalities that could suggest an increased susceptibility to excitatory migraine triggers (88).

The IIB of migraine can also include reduced overall activity, lower levels of mobility, as well as a greater level of sleepiness and reduced vigor when compared to the control subjects (65). Patients with migraine with aura showed executive dysfunction in the interictal phase, and an association between executive disturbances and the duration and intensity of migraine headache (40).

## Emotional and psychological co-morbidities

While the symptoms of migraine are attracting serious research and treatment attention, there are other aspects that are less obvious, and thus less studied. It is becoming clear that the diagnosis of migraine is stigmatizing, which adds to the emotional burden of the patient, and may inhibit seeking treatment (89). For example, historically, migraine had come to be associated with a “sensitive” or “nervous,” personality (25). Even now, people with migraine are often portrayed in media as being lazy, hypochondriac, hysterical, and unable to deal with stress (25). In a study using the validated stigma scale for chronic illness (SSCI), it was found that chronic migraine was as stigmatizing as epilepsy, whereas episodic was less so, and was most highly correlated with ability to work (41). According to an epidemiologic survey that included 9,999 respondents without migraine, 31% believed that patients with migraine use migraine as a way to get out of work or school, 45% believe that migraine is easily treatable, and 36% believe that migraine is a result of unhealthy behaviors (90). Fear of being stigmatized leads to patients being hesitant to seek diagnosis, or to engage in treatment, and adds to the emotional burden of the patient (25). Patients with migraine struggle with the feeling of having an invisible disorder and of being doubted (91). Many patients feel that their migraine is under-recognized and not well-managed due to it being largely attributed to psychological disease (92). It is worth noting that these stigma, descriptors, and misperceptions of people with migraine do not occur only during the migraine attack. These are descriptions that these individuals carry with them at all times, thus adding to IIB.

Along with the stigma associated with migraine come increased risks of psychiatric comorbidities, especially of anxiety and depression, although the causal relationship between these comorbidities and migraine is unclear (26). In a survey conducted in France of patients with migraine and age-matched control subjects, both men and women with migraine showed significantly elevated scores for stress, anxiety, and depression (26). Depressive disorders appear to be the most common psychiatric comorbidity that occurs with migraine, and patients with migraine have ~2–4 times greater odds of developing a depressive disorder sometime during their lifetime when compared to patients without migraine (93). An analysis of results from the large, prospective Women's Health Study found that, among middle-aged women, migraine and non-migraine headache were both associated with an increased risk of incident depression compared to patients who had no history of headache, and that increased headache frequency was associated with a higher risk for developing incident depression (94). In a study that applied polygenic (genetic risk) score analysis, it was found that migraine and major depressive disorder (MDD) are genetically distinct disorders, but that in the subset of subset of migraine patients with MDD, migraine could be either a symptom or consequence of MDD (95). It has also been suggested that shared neurobiology and neurotransmitters may account in part for the association between depression and migraine (92).

In general, it appears that depressive symptoms may accompany migraine episodes; it is reported that they may be preceded by a sense of anxiety (96). Anxiety affects quality of life of patients with migraine, in part because the interictal period is spent in fear, anticipating the next attack (26). Neurophysiologic data supports the association between emotional factors and migraine. For example, dysregulation of the limbic system by the hypothalamus was found in patients with migraine (97), providing a neurologic substrate for the role of stress in migraine (98). In an imaging study of patients with migraine, performed during the interictal period, negative, but not neutral or positive, emotional cues caused the activation of brain regions associated with emotional processing (99). Moreover, there was overlap between regions involved in nociceptive and emotional processing (i.e., posterior cingulate, caudate, amygdala, and thalamus) (99).

## Discussion

The burden suffered by people with migraine encompasses not only the period immediately surrounding the migraine attack but extends throughout the interictal periods. Although symptoms experienced during these phases may be more subtle, they nevertheless impact the quality of life. It is important that patients be encouraged to discuss comfortably their feelings and symptoms even when they may not seem to be directly related to a migraine headache, and for their healthcare providers to actively listen to all patients' concerns.

There is a growing realization that increasing our understanding of IIB as being an important component of migraine deserves considerable attention, as QoL is affected. Moreover, recent studies have now found that not only does the phase between migraine attacks have the potential to have a significant emotional impact on the patient, but it may also include allodynia and hypersensitivity, changes in taste or smell, sensitivity to light or sound, changes in visual perception, and vestibular dysfunctions affecting balance and motion sickness. Collectively, these symptoms contribute to the overall burden experienced by patients with migraine and deserve consideration in research, patient-provider dialogue, and treatment.

The headache burden of migraine is well-documented, and reduction in migraine headache days is a common primary outcome measure that is used in the evaluation of novel preventive therapeutics for migraine. However, as our understanding of the constellation of symptoms associated with migraine throughout the cycle from prodrome through the next prodrome expands, we can appreciate that migraine is much more than the headache phase. Rather, it is a cycling syndrome that exacts a considerable burden on the patient, even independent of the headache itself. Thus, the burden of migraine continues throughout not only the well-examined headache phase, but through the interictal phase as well. The importance of the interictal burden is beginning to receive the attention it requires and is increasingly being included as an outcome measure in clinical trials. There is emerging evidence that suggests that preventive treatments can be beneficial in reducing the interictal burden of migraine, which strengthens the argument for paying more attention to this phase of migraine. However, most studies look only at metrics that pertain to the ictal phases of migraine, notably, numbers of monthly headache days (migraine or otherwise). Consequently, patients who obtain an interictal benefit from treatment may be overlooked. The AHS consensus statement and other publications outline the importance of QoL measures in supporting and continuing treatment of migraine. The IIB is a measurable and important issue that is related to QoL. We propose that all patients should have QoL and IIB assessed in some fashion to make informed treatment decisions and tracking migraine headache days alone does not give an adequate insight into the overall patient journey. Given the importance of the interictal burden for the wellbeing of people with migraine, it is unfortunate that headache diaries fail in capturing this facet of the migraine disease. We hope the greater recognition of IIB as a key component in migraine symptomatology may stimulate further research leading to its incorporation in headache diaries. It is important that patients feel comfortable in expressing their concerns, even when they do not directly relate to the migraine headache. In doing so, they may reveal symptoms that may not be readily apparent in a short interview.

## Author contributions

RAN, MHO, MV, LV, and BBV contributed to the conception of the work, interpretation of data for the work, drafting of the manuscript, and critical revision of the manuscript for intellectual content. All authors agree to be accountable for the content of the work.

## Funding

This work was supported by Eli Lilly and Company.

## Conflict of interest

Authors BBV, LV, MV, and RAN were employed by Eli Lilly and Company and may own some Lilly stock. Author MHO was employed by Evidera.

## Publisher's note

All claims expressed in this article are solely those of the authors and do not necessarily represent those of their affiliated organizations, or those of the publisher, the editors and the reviewers. Any product that may be evaluated in this article, or claim that may be made by its manufacturer, is not guaranteed or endorsed by the publisher.
